# Integrating behavioral health care into a low-barrier HIV clinic using the Collaborative Care Model: a mixed methods evaluation of patient care cascade outcomes and determinants

**DOI:** 10.1186/s43058-025-00738-5

**Published:** 2025-05-05

**Authors:** Scott Halliday, Lydia A. Chwastiak, Kaitlin Zinsli, Ramona Emerson, Teagan Wood, Meena S. Ramchandani, Kenneth Sherr, Judith I. Tsui, Bradley H. Wagenaar, Deepa Rao, Julia C. Dombrowski

**Affiliations:** 1 Department of Global Health, University of Washington, 3980 15 th Ave NE, Seattle, WA 98195 USA; 2 Department of Psychiatry and Behavioral Sciences, University of Washington, Seattle, WA USA; 3 Department of Epidemiology, University of Washington, Seattle, WA USA; 4 Department of Medicine, University of Washington, Seattle, WA USA; 5 Public Health – Seattle & King County HIV/STI/HCV Program, Seattle, WA USA; 6 Department of Industrial & Systems Engineering, University of Washington, Seattle, WA USA

**Keywords:** Collaborative care model, Implementation science, HIV, Depression, Opioid-use disorder, Delivery of health care

## Abstract

**Background:**

Low-barrier HIV care is an evidence-based intervention to improve HIV outcomes among those who have complex barriers to care, but the walk-in model poses challenges to integrating behavioral health services. We evaluated the acceptability and feasibility of a Collaborative Care Model (CoCM) for treatment of depression and opioid use disorder in a low-barrier clinic.

**Methods:**

In a sequential explanatory mixed methods pilot study, we accessed data from patient records to generate a care cascade for the number of patients enrolled in the first six months of the program and conducted individual interviews with patients and staff to interpret the care cascade findings.

**Results:**

Among 175 patients who visited the clinic, 36% were screened for, 24% were referred to, 15% completed an intake for, and 9% engaged in CoCM. The interviews revealed that screening was limited by a lack of clarity among staff about services offered in CoCM, staff forgetting the screening process, and limited time during patent visits. Referrals were limited by low buy-in among staff and patient complexity. Intakes were limited by time and space constraints in the care setting and competing acute patient needs. The care manager’s ability to embody the clinic’s culture facilitated engagement among patients who completed intakes.

**Conclusions:**

Staff perceived CoCM to be acceptable and feasible to implement, but only in the context of multiple barriers to implementation and challenges to systematic screening and measurement-based care.

**Trial registration:**

Not applicable.

**Supplementary Information:**

The online version contains supplementary material available at 10.1186/s43058-025-00738-5.

Contributions to the literature

- We evaluated implementation of the Collaborative Care Model, an evidence-based model for integrating behavioral healthcare into primary care settings, for treating depression and opioid-use disorder in a low-barrier HIV clinic. To our knowledge, this is the first implementation of the Collaborative Care Model in a low-barrier HIV clinic.
- By specifying a multi-component implementation strategy, our evaluation provides a foundation for selecting and tailoring implementation strategies for use with integrated behavioral healthcare interventions.
- Through use of a joint visual display showing a care cascade with illustrative quotes, our evaluation demonstrates a novel approach to using explanatory mixed methods in implementation science.

## Introduction

In the context of improving rates of viral suppression among people with HIV (PWH) in the United States [1] and ongoing initiatives towards Ending the HIV Epidemic [2], those who remain untreated or have suboptimal clinical outcomes often face complex barriers to care and treatment. Mental health and substance use disorders are common among PWH who are not virally suppressed [3, 4] and these conditions are often compounded by poverty, housing instability, incarceration, food insecurity, and a lack of employment [5]. Low-barrier HIV care is a model of care delivery that improves HIV outcomes among PWH with complex barriers who are not well-engaged in traditional models of care [6]. In the Max Clinic (hereafter, Clinic), a low-barrier HIV clinic in Seattle, Washington, only 33% of patients referred for psychiatric services and 40% of those referred for opioid use disorder (OUD) treatment outside of the clinic completed even a single visit [7].

Our team adapted the Collaborative Care Model (CoCM)—an evidence-based intervention for depression [8–10] and OUD [11–13] that uses a task-sharing approach to integrate a non-physician behavioral health (BH) care manager and a consulting psychiatrist into a primary care team—to integrate treatment for depression and OUD into the Clinic and improve the fit of the CoCM model with the low-barrier clinic context [14, 15]. Here we describe the results of CoCM implementation, which has not previously been implemented in a low-barrier HIV care setting, using a mixed methods evaluation. We quantitatively describe the care cascade for patients participating in CoCM and present the results alongside illustrative quotes from qualitative interviews to explain the factors that affected progression of patients through the cascade. We also contextualize these findings by providing a qualitative description of the barriers and facilitators to implementing CoCM in the Clinic.

## Methods

### Study design

We conducted an explanatory, sequential (QUANT- > qual) mixed methods evaluation [16, 17] of patients in the first 6 months of CoCM implementation (May–October 2021; recruitment start and end dates respectively) and tracked their outcomes through the first 12 months of CoCM implementation (May 2021 – April 2022; analysis period). Using data from electronic health records and the CoCM tracking database, we calculated a cascade of progression through the CoCM program, modeled on the widely used HIV care continuum. The care cascade was presented to both patients (*n* = 14) and Clinic staff (*n* = 11) during individual in-depth interviews for interpretation to elicit the elements associated with progression as well as barriers and facilitators to implementation. We included a Standards for Reporting Implementation Studies checklist as Additional file 1, a detailed description of our methods in Additional file 2, and the interview guides in Additional file 3.

### Study setting and patient population

The Clinic receives support from the Washington State Department of Health, the Ryan White HIV/AIDS Program, and the Ending the HIV Epidemic in the US Initiative. Approximately 84% of the Clinic’s patients are Medicaid members, many of whom receive insurance through the Ryan White HIV/AIDS Drug Assistance Program (Clinic program data). The primary feature distinguishing the Clinic from other models of HIV care is that all services, including primary care, are delivered on a drop-in basis (i.e., no scheduled appointments). The Clinic’s model additionally includes integrated high-intensity case management and incentives (e.g., cash, snacks, and food vouchers) for completing visits, laboratory testing, and achieving viral suppression. PWH enrolled at the Clinic have complex medical and social needs; among 357 patients enrolled as of April 2023, 97% experienced housing instability, had a current psychiatric diagnosis(es), and/or indicated substance use (Clinic program data). The Clinic’s team structure, prior to implementation, consisted of infectious disease physicians who were the patients’ primary care providers and delivered team-based primary (and HIV) care; clinical social workers who provided medical case management and assisted with accessing wrap-around supportive services; and public health disease intervention specialists who supported patient navigation, offered psychosocial support, assisted with incentive delivery, and coordinated the Clinic’s patient flow. The Clinic’s model aligns completely with the Substance Abuse and Mental Health Services Administration’s principles and components of low-barrier models of care by including all of the requirements and approach except for extended hours of healthcare delivery [18].

### Recruitment and analysis

For the quantitative assessment of the CoCM care cascade, all patients enrolled in the Clinic were included based on EHR and program data. This included the number of patients with ≥ 1 visit to the Clinic during the analysis period, and who were screened for CoCM, referred to CoCM, completed intake (≥ 1 CoCM encounter), and engaged in (≥ 2 encounters) COCM. Patients were excluded from the study if they had a schizophrenia spectrum diagnosis, less than six months life expectancy, or were already engaged in treatment for depression and/or OUD elsewhere.

For the qualitative interviews, we exhaustively recruited staff (13 total staff with 2 declining) working in the Clinic during CoCM implementation, while patients enrolled in the Clinic were sampled conveniently during a walk-in visit from among a purposive stratified (by steps of the CoCM cascade) sampling frame [19]. The interviews were conducted by research team members not involved in CoCM implementation, audio-recorded and transcribed, and thematically analyzed using inductive and deductive coding techniques [20, 21]. For defining the barriers and facilitators to implementing CoCM at the Clinic, we used questions with optional probes that were associated with the anticipated determinants we identified in the formative evaluation as informed by the 2009 version of the Consolidated Framework for Implementation Research [22, 23]. Briefly, these included: relative advantage (intervention characteristics), patient needs & resources (outer setting), compatibility, relative priority, and available resources (inner setting).

Staff received a $20 gift card and patients received $50 cash in compensation. This study was approved by the Human Subjects Division at the University of Washington (STUDY00010501).

### Intervention

As described in detail elsewhere [15], the process of adapting CoCM followed interviews with patients and service-delivery stakeholders. The core CoCM components (patient-centered team care; evidence-based psychosocial and pharmacologic treatment; care management using an electronic health record (EHR); and weekly case review meetings with the psychiatric consultant, the care manager, and a primary care physician) were maintained, [24, 25] while adaptable aspects of the intervention were iteratively refined. The care manager was a dedicated, part-time, and co-located registered nurse at the Clinic with experience in outpatient BH clinical settings (including in adjusting dosage of buprenorphine) who received three months of protocolized training in CoCM (see Table 1 below). The consultant psychiatrist was based at the same hospital and was available for remote consultation with the Clinic staff as needed in addition to the weekly case review meetings. In addition to the protocolized training for the care manager, all Clinic staff were onboarded to CoCM via small group and large group consensus discussions, where the staff roles with respect to screening, referral, intake, and other logistical processes (e.g., assigning rooms, handling cases when care manager was absent, and facilitating hand-offs between different Clinical staff) were iteratively refined (see Table 1). In addition, increased flexibility around care manager clinical contacts was introduced: PHQ- 9 measurement was not required at every contact and contacts were not expected to occur with regular frequency.

**Table 1:** Specifying the multi-component Advancing Integrated Mental Health Solutions implementation strategy for the CoCM

Domain	Involve patients & stakeholders	Local consensus discussions	Technical assistance	Care manager training	Visiting other sites^a^	Conduct small tests of change
Definition	Individual in-depth interviews to identify anticipated barriers and facilitators to implementation	Small and large group meetings on implementation decisions, planning adaptations, and staff buy-in	Longitudinal supervision for the care manager and ongoing implementation support for delivery of the CoCM	A multi-component, protocolized training for the care manager in delivery of the CoCM	Care manager visited clinics implementing the CoCM to establish a professional network and to observe the CoCM	Interactive problem-solving process to adapt the CoCM to improve its contextual fit at the Clinic
Actors	Research team^b^	Research team^b^	Research team and external partners^b^	Research team and external partners^b^	Research team and external partners^b^	Research and small group consensus team^b^
Actions	Collect and analyze data and prepare discussion points for consensus discussions	Hold meetings and operationalize feedback into implementation plan	Use evidence, experience, and data to address implementation challenges	Combination of self-paced online didactic training sessions and 1:1 skill building sessions.^3^	Care manager visited clinics implementing the CoCM to observe other implementers and case reviews	Continuous quality improvement to respond to BH care manager, staff, and patient feedback
Action target	Patients and Clinic stakeholders	Clinic stakeholders	Care manager and Clinic stakeholders	Care manager	Care manager	Clinic stakeholders
Temporality	Pre-implementation	Pre-implementation	Pre-implementation and implementation	Pre-implementation and implementation	Pre-implementation	Implementation
Dose	One 30–60-min interview with each stakeholder and each purposely selected patient.^d^	Small group: three 1 h meetings Large group: two 30 min presentations	Weekly 60-min meetings in early implementation phase; decreasing frequency later	Approximately three months of training in total.^c^	Three half day visits to other Max Clinic clinics with the CoCM	Two quality improvement cycles resulted in targeted universal screening and adjusted care plans for patients
Implementation outcomes targeted	Feasibility	Acceptability, feasibility, appropriateness	Fidelity, penetration	Fidelity	Fidelity	Feasibility, penetration, sustainability
Justification	All components of the multi-component Advancing Integrated Mental Health Solutions implementation strategy for the CoCM were iteratively developed in conjunction with 20 years of implementation experience in response to implementation-related challenges. An observational study found that compared to low or basic levels of implementation support, healthcare settings implementing the CoCM for people with depression using the multi-component Advancing Integrated Mental Health Solutions implementation strategy had better health outcomes [26]					

^a﻿^Included the Adult Medicine Clinic and the Mental Health and Addiction Services Clinic at Harborview Medical Center

^b﻿^Research team includes Principal Investigator JD, study team University of Washington-affiliated co-investigators (LC, KS, JT, DR, Research Assistant (SH), and BH care manager (RE). External partners include other stakeholders affiliated with the site clinics visited by the BH care manager and/or stakeholders affiliated with the University of Washington Advancing Integrated Mental Health Solutions Center, which developed the training materials. Representatives from small group consensus meetings include 1 member from each care delivery team employed with the Max Clinic: disease intervention specialists, social workers (TW), and physicians (MR); a research team member (LC); the Research Assistant (SH); and the BH care manager (RE)

^c^Includes: self-paced, 6–8 hour online, didactic Advancing Integrated Mental Health Solutions Center course on epidemiology of depression, the CoCM’s evidence base, team member roles, and CoCM components; 1:1 skill development sessions with research team on registry, measurement-based care, systematic case review, and Behavioral Activation; 6 hour suicide prevention course offered by University of Washington Department of Psychiatry and Behavioral Sciences for Washington State physicians; a refresher training in office-based opioid treatment; psychological intervention modules with a focus on motivational interviewing and distress tolerance; and overview of HIV treatment module

^d^See Study Populations and Recruitment for details of the sampling process

The structure of low-barrier care (e.g., lack of scheduled appointments), the Clinic’s team structure, the patient populations’ complex needs, and time constraints necessitated changes to the population-based screening for depression and OUD. Screening for depression and OUD initially targeted only patients who were assessed by the clinical social workers, but this was later changed to a staged universal screening for CoCM conducted by all Clinic staff. The Clinic staff used the Patient Health Questionnaire (PHQ)− 2 followed by the PHQ- 9, if indicated and the National Institute for Drug Administration (NIDA) Quick Screen, which was modified to include opioid use.

For patients with depression, following the intake step, the patient’s primary care provider at the Clinic prescribed medication and the care manager offered Behavioral Activation as a brief, evidence-based psychotherapy [27] and assisted with adjusting medication based on recommendations from the consulting psychiatrist and the patient’s primary care provider during the following clinical contacts. For patients with OUD, following the intake step, the patient’s primary care provider would prescribe buprenorphine and the care manager adjusted the dosage during the following clinical contacts.

### Implementation strategy

To facilitate implementation of the adapted CoCM model in the Clinic, we used a multi-component implementation strategy created by the University of Washington Advancing Integrated Mental Health Solutions Center [25]. Table 1 specifies the strategy’s components [28].

## Results

### CoCM care cascade

**Fig. 1:**
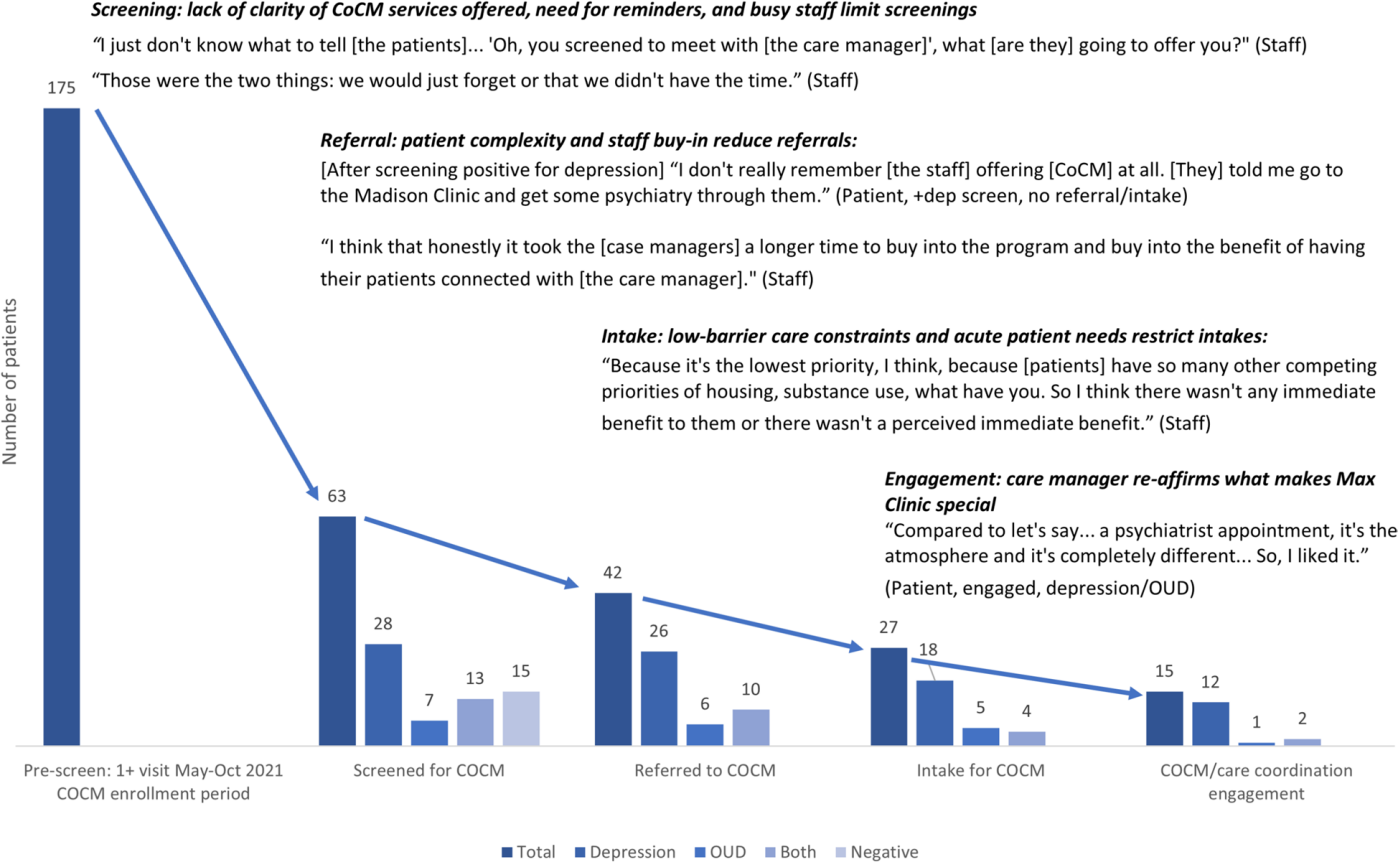
CoCM low-barrier HIV care cascade with interpretive quotes and summaries of factors associated with progression. Each number represents the total number of patients at each cascade step and each step is denominator-denominator linked [29]. We define the engagement step as initiating direct care services with the BH care manager (including starting or adjusting an antidepressant medication and/or psychotherapy for patients with depression or starting a medication for OUD for patients with OUD) and/or coordinating BH care with another provider. Six people who screened positive were not referred for an intake: four declined an offer for an intake and two were not referred by the screener

During the analysis period, 175 patients completed ≥ 1 visit to the Clinic during the 6-month analysis period (Table 2). Of these, 38% [67] had a chart diagnosis of major depressive disorder only, 20% [17] had diagnosis of opioid use disorder only, and 27% [48] had both diagnoses. As shown in Fig. 1, 63 (36%) of patients were screened for CoCM, 42 (24%) were referred, 27 (15%) completed an intake and 15 (9%) engaged in CoCM.

**Table 2:** Descriptive characteristics of patients

	Overall (*N* = 175)^a^
**Gender**	
Cisgender man	117 (66.9%)
Cisgender woman	41 (23.4%)
Nonbinary, Genderqueer, or Other	3 (1.7%)
Transgender woman	8 (4.6%)
Unknown or not disclosed	6 (3.4%)
**Age**	
Less than 30 years	35 (20.0%)
30–39 years	54 (30.9%)
40–49 years	64 (36.6%)
50–59 years	19 (10.9%)
60 years or older	3 (1.7%)
**Race and Ethnicity**	
Black	44 (25.1%)
Hispanic	16 (9.1%)
Other	23 (13.1%)
White (non-Hispanic)	92 (52.6%)
**Housing Status**^**b**^	
Stable	51 (29.1%)
Unstable	124 (70.9%)
**Injection Drug Use**^**c**^	
Injection drug use	72 (41.1%)
No injection drug use	79 (45.1%)
Missing	24 (13.7%)
**Substance Use**^**c**^	
Methamphetamine	133 (76.0%)
Heroin	65 (37.1%)
Cocaine or crack cocaine	42 (24.0%)
Hazardous alcohol use	30 (17.1%)
Marijuana	86 (49.1%)
Prescription-type opioids	2 (1.1%)
Benzodiazepine	13 (7.4%)
No substance use	12 (6.9%)
**Psychiatric diagnoses**^**c**^	
Depression	115 (65.7%)
Bipolar or related disorders	35 (20.0%)
Anxiety	64 (36.6%)
Post-Traumatic Stress Disorder	40 (22.9%)
Schizophrenia spectrum or other psychotic disorders	46 (26.3%)
Neurodevelopmental disorders (excluding ADHD)	4 (2.3%)
Attention Deficit Hyperactivity Disorder	12 (6.9%)
Personality disorders	10 (5.7%)
Feeding and eating disorders	4 (2.3%)
No psychiatric diagnosis	25 (14.3%)

^a^Measured approximately one year prior to the May–October 2021 enrollment period via chart review. Note: substance use indications and psychiatric diagnoses may thus differ from the results of the CoCM screening process

^b^Unstable housing includes: sleeping outside, staying in a shelter, transitional housing (e.g., medical motel), or couch-surfing

^c^Not mutually exclusive as patient may endorse using multiple substances and/or have multiple psychiatric diagnoses

### Interview themes related to the CoCM care cascade

We conducted interviews with 14 patients (none declined) and 11 staff (2 declined). Key themes with illustrative quotes for each step are included in Fig. 1 and summarized below.

#### Screening: low priority during limited time with patients and lack of clarity about services offered

Some staff reported forgetting to complete the screening or de-prioritizing it when there were competing demands on their time. The screening process was perceived as conflicting with the typical visit to the Clinic, which is structured around a patient’s goals and is often shorter than visits in other primary care settings. “I think for some of the patients that just wanted [to quickly] get out the door and then we’re like, ‘Oh, hey, I have this screening to go through with you.’ It’s not the thing that they’re wanting to do because they just were not anticipating that” (Staff).

Social worker and physician knowledge of patients’ comorbidities played an important role in uptake of screening. Care team members expressed reluctance to systematically screen for depression and OUD when they knew the patient would not be eligible for CoCM due to having psychosis symptoms or diagnosis. This shaped the decision to adapt the proactive and systematic screening for depression and OUD to an assessment focused on eligibility for CoCM. Additionally, staff expressed confusion over the screening process and what being referred to CoCM would entail for their patients.

*I think [it] became clear, after we talked about it a couple times at staff meeting, was whether or not people qualified for the program. If they had other diagnoses that weren’t just depression or opioid use disorder. So, I think a lot of people who have either a poly-substance use diagnosis or another primary psychiatric diagnosis, or who used meth, which is a lot of people, didn’t necessarily qualify* (Staff).

#### Referral and intake: staff buy-in, lag between referral and intake, and low-barrier constraints

Of the 63 who screened, 42 screened positive and were referred, 15 screened negative and were not referred, 4 screened positive but declined a referral, and 2 screened positive but were not referred by staff for unknown reasons. One issue affecting referral was lack of staff buy-in to the CoCM program. Some social workers had experience providing BH services, but their role at the clinic was more specific to medical care coordination and linking patients to wrap-around social services (e.g., finding stable housing). For some, their experience with BH services led to skepticism about having a nurse in a care manager role.

It is notable that most patients referred to CoCM did not complete a same-day intake visit. But even patients who accepted the idea initially frequently expressed a preference to return for an intake on a different day (but who rarely completed the intake). Between visits, which could span weeks or months, some patients’ BH needs and priorities changed, some lost interest in CoCM, and others did not remember being referred to CoCM. This last reason was mentioned as a factor by one patient who did not complete the intake but nonetheless expressed continued interest. On whether they’d prefer co-located BH at the clinic vs referral to an external provider: “Yeah, that would be easier and better… I would enjoy that very much so” (Patient, referred for depression but no intake).

Responding to a patient’s willingness to engage in BH care is time-sensitive and any delay in connecting them with a provider can lead to disengagement. At the start of CoCM implementation, the care manager was unable to meet all interested patients due to time constraints. “… At first… she was only here… two days a week and then eventually it increased to three days. And I think the three days a week helped” (Staff). Additionally, due to the walk-in nature of the clinic and the incentivized care delivery, patients may not have the expectation of seeing a care manager during a visit.

*People would often come in just to get their incentives. And then [the care manager] would be like, “Hey, do you want to meet”… I mean, I don't go to the doctor every two weeks, but every time I went… somewhere I go regularly and my therapist jumped out and was like, “Hey, do you want to talk?” I feel like that might feel… a little bit intrusive or you're just like, “no, not right now. I came in, I was expecting this to take 10 minutes”* (Staff).

#### Engagement: care manager models the clinic culture and values

For the patients who were engaged in CoCM, the care manager’s ability to deliver care consistent with the values that make the Clinic special was essential. By bringing BH on-site in the Clinic, patients and staff expressed that the services were more accessible and welcoming with one staff member remarking: “I think [the care manager] was super approachable” (Staff).

Some engaged patients felt the care manager’s support and the CoCM program helped them to better adhere to taking HIV medications. “I was really bad at taking my [HIV] meds. I just didn't really see a point to it. And with all of the [CoCM] services you guys provide here, it made it really easy to start caring again… So, I'm undetectable now” (Patient, engaged, depression).

### Observed barriers and facilitators to implementing CoCM

We identified multiple barriers and facilitators to implementing CoCM, which are summarized using the Consolidated Framework for Implementation Research in Table 3. We provide illustrative quotes and contextual descriptions in Additional File 4. Briefly, the barriers included staff reluctance about screening, which intersected with the culture of low-barrier care and knowledge of patient BH needs; negative perceptions of time and resource constraints impacting different steps of the care cascade; and the care manager’s low self-efficacy to provide Behavioral Activation coupled with the poor fit of Behavioral Activation with low-barrier care. The care manager modeled the Clinic’s organizational culture – which enhanced patient engagement – was a facilitator to implementation.

**Table 3:** Observed determinants of implementing CoCM in low-barrier HIV care

Domain	Construct	Sub-Construct	Observed barriers (-)/facilitators (+)	
			Patients	Stakeholders
**Intervention Characteristics**	Evidence Strength and Quality			+ / -
	Relative Advantage		+	+
	Adaptability			+
	Trialability			+
	Complexity			-
**Outer Setting**	Patient Needs & Resources			-
**Inner Setting**	Networks and Communication			+ / -
	Culture		+	+
	Implementation Climate	Compatibility		+ / -
	Readiness for Implementation	Available Resources	+	-
**Characteristics of Individuals**	Self-Efficacy			-
	Individual Identification with Organization		+	+
	Other Personal Attributes		+	+
**Process**	Reflecting and Evaluating			+

### Factors associated with sustaining CoCM

While staff held mixed opinions on whether CoCM should be sustained at the Clinic, we identified several factors (see Additional file 4) that may enhance CoCM’s sustainability. These included practical adjustments to the screening process, increasing human and time resources, offering multiple types of therapy, and improving intra-team communication.

## Discussion

In this sequential explanatory mixed-methods evaluation of integrating care for depression and OUD in a low-barrier HIV clinic, we found that CoCM was acceptable and feasible to implement but only in the context of multiple barriers to implementation and challenges to systematically screening patients and providing measurement-based care in the low-barrier setting. Whereas most parts of the multi-component implementation strategy focused heavily on training the care manager in delivering CoCM, there was less intentional emphasis placed on generating staff buy-in and clearly protocolizing all steps of the screening, intake, and referral processes. PWH who have depression and/or OUD may be doubly (or triply) stigmatized and may face additional barriers to accessing care. In this context, we found that a BH program co-located within the primary care setting with a non-physician care manager can meet patients where they are [30]. Reconciling these limitations, iteratively adapting CoCM to improve its contextual fit at the Clinic, and supporting high quality intra-team communication among Clinic staff will be essential to sustaining CoCM.

Although our care cascade lacked subsequent steps evaluating individual patient outcomes, our results for those with depression compare favorably to other studies. Cholera and colleagues constructed a care cascade for PWH and depression, and observed that among those, 43% received antidepressants [31]. Similarly, Pence and colleagues estimate that 40% of those clinically diagnosed with depression received any treatment [32]. Here, we observed that 42% of patients who screened positive for depression and 67% of patients completing an intake for depression care through CoCM were engaged in care. Limited care cascade data exist for interventions integrating care for OUD among PWH to our knowledge. In a prospective cohort study offering opioid agonist therapy for PWN and OUD in Vancouver, Canada, 80% of participants who endorsed using opioids daily at study baseline and who had at least one study visit during the enrollment period were on opioid agonist therapy [33] whereas we observed only a single engaged OUD patient from among seven who endorsed using opioids (14%).

Our study has several limitations. In this pilot study, CoCM was implemented as standard-of-care and there was no comparison group. For the qualitative strand of this study, due to time and resource constraints, we were unable to use multiple coders in the analysis, which potentially limited the depth, diversity, and credibility of the findings. Administrative policies required that all patient interviews were performed in patient examination rooms, which may have influenced responses. For the quantitative strand of the study, despite the initial intent to collect patient clinical outcomes towards assessing effectiveness, these data were not routinely collected as the care manager focused heavily on working to engage patients. Pre-screening diagnoses and substance use endorsements were captured from electronic health records and social worker assessment prior to the start of CoCM, and may not have reflected the true status of patients at the time of consideration for CoCM. Lastly, due to the study’s focus on acceptability and feasibility and the care manager’s focus on working to engage patients in care, we do not have quantitative data (and limited qualitative data) on the quality of care in CoCM.

## Conclusion

In this sequential explanatory mixed methods evaluation of implementing CoCM in a low-barrier HIV setting for depression and OUD, we observed that 36% of patients were screened for CocM, 24% were referred to CoCM, 15% completed an intake for CoCM, and 9% progressed to the engaged step of the CoCM care cascade. The explanatory elements associated with progression through these respective steps included: for screening, confusion among stakeholders about CoCM, staff forgetfulness to do the screening in the context of competing demands, and brief patient visits hindered staff ability to complete screenings; for referral, poor staff buy-in and the complexity of patient BH needs; for intake, the limited time, space, and human resources associated with low-barrier care as well as patients with acute medical and socio-economic needs; and for engagement, the BH care manager successfully exemplified the Clinic’s unique culture and values. Our findings indicate that while CoCM was acceptable and feasible to implement at the Clinic, it was so only in the context of multiple observed barriers to implementation and challenges to systematically screening patients and providing measurement-based care in the low-barrier setting.

## Supplementary Information

Additional file 1. Standards for Reporting Implementation Studies: the StaRI checklist for completion.

Additional file 2. Detailed study methods description.

Additional file 3. Data collection instruments.

Additional file 4. Assessment of barriers and facilitators to implementing CoCM and factors associated with sustaining CoCM.

## Data Availability

De-identified qualitative transcripts, memos, coding notes, and quantitative data are not publicly available as they contain quotes and information that could compromise participant identities. The risk of re-identification is high considering the research was done at a single site with a small total population of actively enrolled patients or clinic stakeholders. Due to the stigma associated with living with HIV and co-occurring behavioral health conditions, these data are also more sensitive. Requests for data may be sent to: jdombrow@uw.edu. Statistical software was used minimally for cleaning the descriptive quantitative data and preparing tables, but not for the descriptive quantitative analysis, which was carried out in Excel. Reproducible code is available upon request by emailing: jdombrow@uw.edu.
