# Supplementary material for: Integrating behavioral health care into a low-barrier HIV clinic using the Collaborative Care Model: a mixed methods evaluation of patient care cascade outcomes and determinants
Source: Implement Sci Commun. 2025 May 5;6:53. doi: 10.1186/s43058-025-00738-5 (PMC12053849; doi:10.1186/s43058-025-00738-5)
Supplement: Supplementary file 2 — Additional file 2. Detailed study methods description. [file 43058_2025_738_MOESM2_ESM.docx]

*Additional File 2: Detailed study methods description*

*Data collection*

To describe the Collaborative Care Model (CoCM) cascade, we extracted patient and demographic data from the Max Clinic EHR system and linked these with CoCM programmatic records maintained in a REDCap database (1, 2). The REDCap database contained data collection tools for standardized screening for depression (PHQ-2 followed by the PHQ-9, if indicated) and OUD (National Institute for Drug Administration (NIDA) Quick Screen, modified to include opioid use). The CoCM intake process additionally included the Generalized Anxiety Disorder 7-item (GAD-7) and questions about HIV viral load, previous diagnoses of other severe mental illnesses (dementia, severe cognitive impairment, and/or psychosis and schizophrenia spectrum), opioid use in the past 90 days, life expectancy, and an imminent safety risk assessment. For patients engaged in CoCM, the REDCap instrument contained the following: the PHQ-9; GAD-7; questions for medications for OUD monitoring, and an indicator to flag cases for review in the weekly case discussion.

We developed separate interview guides (see Additional File 4) for semi-structured, individual in-depth interviews with patients and staff. Each guide contained questions about the participant’s general experience with CoCM, factors associated with each step of the care cascade, the barriers and facilitators to implementing CoCM, and factors influencing sustainability or scale-up of CoCM. The interview guides also included probes informed by the Consolidated Framework for Implementation Research qualitative interview guide that could be used as needed to explore relevant domains and constructs based on the most salient contextual factors that were previously identified during the formative research study.(3) The care cascade was presented to both patients and staff members during individual in-depth interviews to elicit perceptions of the elements associated with progression. Patient interview questions were adjusted based on their level of involvement with CoCM. Patient interviews were held in the clinic and staff interviews were held by videoconference or in-person; all were recorded and transcribed.

*Analysis*

We determined the number of patients with ≥1 visit to the Max Clinic during the analysis period, and within this group, the number and proportion of people who were screened for CoCM, referred to CoCM, and completed an intake visit for CoCM. We defined engagement in CoCM as having ≥2 encounters with the BH care manager for mental health treatment, OUD treatment, or care coordination. For thematic analysis of the interviews, we used both deductive and inductive coding techniques. SH singly coded all interviews, keeping memos, tracking emergent themes as coding progressed, and annotating codebook changes. SH was a graduate student in implementation science; had experience with implementing CoCM in multiple settings; used the Consolidated Framework for Implementation Research as a tool to guide qualitative data collection and for thematic analysis to identify barriers and facilitators to implementation; and was trained in qualitative methods with a focus on semi-structured individual in-depth interviews with patients and staff, thematic analysis, and both inductive and deductive coding to identify barriers and facilitators to implementing evidence-based programs. For identifying the factors associated with progression through the care cascade, we started with *a priori* code groups associated with each cascade step and then used inductive coding techniques to iteratively refine the codebook. For defining the barriers and facilitators to implementing CoCM at the clinic, we used deductive coding with imported codes associated with the anticipated determinants identified in the formative evaluation as informed by the Consolidated Framework for Implementation Research (3, 4). To explore factors associated with sustaining CoCM, we used an inductive coding approach. We achieved meaning saturation for identifying factors associated with progression across the care cascade after approximately half of the staff interviews and three quarters of the patient interviews and for identifying barriers and facilitator after approximately three quarters of the staff interviews (5). All qualitative analyses used Dedoose version 9.0.83 (6)

*References*

1. Harris PA, Taylor R, Thielke R, Payne J, Gonzalez N, Conde JG. Research electronic data capture (REDCap)—A metadata-driven methodology and workflow process for providing translational research informatics support. J Biomed Inform. 2009;42(2):377-81.

2. Harris PA, Taylor R, Minor BL, Elliott V, Fernandez M, O'Neal L, et al. The REDCap consortium: Building an international community of software platform partners. J Biomed Inform. 2019;95:103208.

3. Damschroder LJ, Aron DC, Keith RE, Kirsh SR, Alexander JA, Lowery JC. Fostering implementation of health services research findings into practice: a consolidated framework for advancing implementation science. Implement Sci. 2009;4(1):50.

4. Halliday S, Dombrowski JC, Emerson R, Beima-Sofie K, Chwastiak LA, Sherr K, et al. Formative qualitative research to guide implementation of the Collaborative Care Model in a low-barrier HIV clinic. AIDS Care. 2024:1-14.

5. Hennink MM, Kaiser BN, Marconi VC. Code Saturation Versus Meaning Saturation: How Many Interviews Are Enough? Qual Health Res. 2017;27(4):591-608.

6. Dedoose Version 9.0.83, web application for managing, analyzing, and presenting qualitative and mixed method research data. Los Angeles, CA: SocioCultural Research Consultants, LLC; 2023.
