# Supplementary material for: Integrating behavioral health care into a low-barrier HIV clinic using the Collaborative Care Model: a mixed methods evaluation of patient care cascade outcomes and determinants
Source: Implement Sci Commun. 2025 May 5;6:53. doi: 10.1186/s43058-025-00738-5 (PMC12053849; doi:10.1186/s43058-025-00738-5)
Supplement: Supplementary file 3 — Additional file 3. Data collection instruments. [file 43058_2025_738_MOESM3_ESM.docx]

*Additional File 3: Data collection instruments*

Mixed methods evaluation – Interview Guides

Implementation Study of Collaborative Care Management in the Max Clinic

Post-Implementation Patient Qualitative Interview Guide

Version Date: 03.17.2022

Notes:

Qualitative transcripts to be stored in REDCap during transcription/coding as audit trail in case of accidental deletion

Introduction

***If participant did not participate in pre-implementation interview, share brief introduction of interviewer’s role.

Let me begin by telling you a little about the purpose of this research and how this interview will go. I’ll offer a chance for you to ask any questions, and then we will get started with the discussion. Now that the Collaborative Care Model program [behavioral healthcare nurse program] has been running, we want to: understand what patients think about the program so far and discuss the future of the program.

I want to make sure that I truly hear and understand what you say today. I brought a device to record our conversation so that I can go back and listen to it. The only people who will be able to hear this recording are people on my team who are directly involved with this project and someone who is paid to type up our conversation. The recording will be deleted after my team has analyzed the results from the interviews that we conduct. I might also take some notes as we talk. To help make sure that your privacy is respected, I will use only your first name only if needed. If I ask a question that you don’t want to answer, please feel free to say “pass”.

Do you have any questions at this point? (Wait and respond to any questions.)

Ok, I’m going to start the recorder now (turn on recorder).

Grand tour/opening:

1. Can you please share your experiences receiving care for [depression/opioid-use disorder] at the MAX clinic with the behavioral healthcare nurse?
   1. Probe: what services did you receive? Were you:
      1. Screened by a social worker or a disease research intervention specialist [non-nurse staff member] for depression and/or opioid use disorder? What did you think of the screening process?
         1. Do you remember these 9 questions [have a PHQ-9 screener]? What did you think at the time?
         2. Do you remember being asked about substance use [have a NIDA quick ASSIST]? What did you think at the time?
      2. Referred to the behavioral healthcare nurse for an intake? What did you think of the intake process?
      3. Did you have phone calls or text conversations with the behavioral healthcare nurse about your behavioral healthcare needs? What did you think of these interactions?
      4. Did you start and/or adjust a new medication and/or receive therapy from the behavioral healthcare nurse? What did you think of this care?
   2. Probe: what impressions do you have about the behavioral healthcare you have received?
   3. Probe: what about the behavioral healthcare services have gone well for you? What could be improved?
2. How have your experiences with care compared with any past care you have received elsewhere for [depression/opioid-use disorder]? any thoughts, reflections, or impressions you have about how the Collaborative Care Model [behavioral healthcare nurse] has been running so far?

Interpretive evaluation:

1. Which of these parts of the program have been helpful for you? How have they been helpful to you?
   1. Probe: screening
   2. Probe: intake
   3. Probe: communication and care coordination with behavioral healthcare manager (texts, phone calls, etc)
   4. Probe: care coordination with other behavioral healthcare providers
   5. Probe: services offered by behavioral healthcare providers (medication management, therapy).
2. What factors [in your life>when at the clinic] that have influenced your ability to engage in these services?
   1. Probe: what are the things that are important to you when you arrive at the clinic?
   2. Probe: please help me understand your state of mind when you attend the clinic.
   3. Probe: how did your expectations for seeing the behavioral healthcare manager compare with what was offered to you/what you experienced?
   4. Probe [drop-out at various steps]: what were the reasons for why you attended clinic but declined to see the behavioral healthcare nurse when they were available?
   5. Probe: what has helped you stay engaged [things that the MAX clinic has done vs. outside the MAX clinic]?
   6. Probe: what has hindered your ability to stay engaged?
   7. Probe: what would help you overcome any challenges associated with staying engaged?
   8. Probe: if and how have these factors varied at each step of screening>intake>care coordination and services?
   9. Probe: what else could we be doing to improve your engagement?
3. Would you like to continue receiving care for [depression/opioid-use disorder] at the MAX clinic? Should we continue offering this program?
   1. Probe: what parts of the program would you like to keep the same and why?
   2. Probe: what parts of the program would you like to change and why?
4. Thinking of all the things in your life that you need to deal with How important for you is receiving care for [depression/opioid-use disorder] at the MAX clinic?
   1. Probe: medication vs. therapy vs. coordination.

Implementation Study of Collaborative Care Management in the Max Clinic

Post-Implementation Service Delivery Stakeholder Qualitative Interview Guide

Version Date: 03.17.2022

Notes:

Qualitative transcripts to be stored in REDCap during transcription/coding as audit trail in case of accidental deletion

Introduction

Thank you for participating in this interview.

***If participant did not participate in pre-implementation interview, share brief introduction of interviewer’s role.

Let me begin by telling you a little about the purpose of this research and how this interview will go. I’ll offer a chance for you to ask any questions, and then we will get started with the discussion. Now that the Collaborative Care Model program [behavioral healthcare nurse with consulting psychiatrist] has been running, we want to: understand what Max Clinic staff members [service delivery stakeholders] think about the program so far, receive assistance in interpreting our findings, and discuss the future of the program.

I want to make sure that I truly hear and understand what you say today. I brought a device to record our conversation so that I can go back and listen to it. The only people who will be able to hear this recording are people on my team who are directly involved with this project and someone who is paid to type up our conversation. The recording will be deleted after my team has analyzed the results from the interviews that we conduct. I might also take some notes as we talk. To help make sure that your privacy is respected, I will use only your first name only if needed. If I ask a question that you don’t want to answer, please feel free to say “pass”.

Do you have any questions at this point? (Wait and respond to any questions.)

Ok, I’m going to start the recorder now (turn on recorder).

Grand tour/opening:

1. Can you please share any thoughts, reflections, or impressions you have about how the Collaborative Care Model has been running so far?

Interpretive evaluation:

Here is a care cascade showing how patients are flowing through the program from screening through intake, and various care engagement strategies. We want to understand how to increase retention and improve engagement at all steps. What do you think accounts for the drop-offs we are seeing at these various steps?

1. Some referred patients have had 1+ visits to the MAX Clinic since referral but have not enrolled in the CoCM program. What are the reasons these patients have not followed-up with Ramona?
   1. Probe: what strategies should we use to address these reasons?
2. For the screening process, we initially did a targeted screening approach using the social workers before referral to the behavioral health care manager. Then we adjusted to allow the disease research intervention specialists to also screen as part of a staged universal screening. What are the reasons that some staff members declined to screen patients for the Collaborative Care Model program?
   1. Probe: differentiating between targeted social worker screening vs. disease research intervention specialist staged universal screening?
   2. Probe: reasons patients decline the initial screening?
3. How have the different components of the screening processes gone so far?
   1. Probe: what do you think about the use of the PHQ-2>PHQ-9 for depression screening?
   2. Probe: what alternatives might we employ for screening for depression or identifying patients for the Collaborative Care Model program?
4. Let’s examine the care cascade comparing disease condition (depression vs. OUD). What are the reasons explaining these differences?
5. What do you think have been the successes and challenges associated with the behavioral health care manager role at the MAX clinic?
   1. Probe: integration within team structure?
   2. Probe: communication and workflows?
   3. Probe: qualifications/background nurse vs. licensed clinical social work vs. other cadres of behavioral healthcare workers (licensed counselors)
6. We want to get your opinions as to how the Collaborative Care Model is or is not working to improve patient outcomes on viral suppression. What has been the effect, if any, of the Collaborative Care Model on rates of viral suppression?
   1. Probe: how has Collaborative Care affected rates of viral suppression?
   2. Probe: which parts of Collaborative Care are important (or not) in affecting rates of viral suppression?
      1. Task-shifted care by behavioral health care manager
      2. Services provided by behavioral health care manager (behavioral activation, care coordination, etc)
      3. Case review/case conference with consulting psychiatrist
      4. Registry
7. We used different strategies to improve the implementation of Collaborative Care including training, conducting small group consensus meetings, and involved patients and staff members. Did these help with implementation, and if so, how?
   1. Probe: what other strategies could be helpful?
      1. Cash incentive for following-up with the behavioral healthcare nurse or other form of engagement with the behavioral healthcare nurse?
   2. Probe: in what ways could we have adjusted the strategies we used?
   3. Probe: did these improve the acceptability or feasibility of Collaborative Care? If so, how?
8. What are the other priority behavioral health conditions among patients receiving care at the MAX Clinic?
   1. Probe: how could we more effectively address these conditions?
   2. Probe: what else is needed in the community or outside the clinic to support the behavioral healthcare needs of MAX Clinic patients [CoCM: depression/opioid-use disorder vs. other behavioral health needs]?

Determinants of implementation:

1. Prior to implementation, we identified potential barriers and facilitators to implementing Collaborative Care which included: openness and optimism for Collaborative Care, the MAX Clinic has a culture of solving for the patient but sometimes at the expense of detailed planning, space/room constraints, and questions about role clarity BH Care Nurse<>SW<>DRIS. What have been the barriers or facilitators to implementing Collaborative Care so far [what parts of Collaborative Care have been challenging/easy to implement?]?

CFIR Interview Guide Probes [available for use as needed]

Relative Advantage:

1. How does the intervention compare to other alternatives that may have been considered or that you know about [standard of care/referral to Madison Psyc./HMHS/other]?
   1. Probe: What advantages does the intervention have compared to these other programs?
   2. Probe: What disadvantages does the intervention have compared to these other programs?

Patient Needs & Resources:

1. How well do you think the intervention met the needs of the individuals served by the MAX Clinic?
   1. Probe: In what ways did the intervention meet their needs? E.g. in terms of access to services? Wait times? Linkage to care? Engagement in behavioral/HIV care? Self-management?
   2. Probe: How have past patient experiences receiving behavioral healthcare services affected their participation in Collaborative Care?
2. How do you think the individuals served by the MAX Clinic responded to the intervention?

Compatibility

1. How well did Collaborative Care fit with existing work processes and practices at the MAX Clinic?
   1. Probe: What issues or complications arose?

Relative Priority

1. How does the priority of implementing Collaborative Care compare with other priorities at the MAX Clinic?

Available Resources

1. Did the MAX Clinic have sufficient resources to implement and administer Collaborative Care?
   1. Probe: Were space/room constraints a factor?
   2. Probe: Were waiting room waits a factor?

Sustainability

1. Do you think we should offer Collaborative Care permanently at the MAX Clinic?
2. What adaptations or changes need to be made to facilitate offering Collaborative Care permanently?
   1. Probe: changes to the MAX Clinic
   2. Probe: changes to the Collaborative Care program
3. What additional factors would influence the sustainment of Collaborative Care permanently at the MAX clinic (as opposed to the pilot program?)
   1. Probe: what factors would influence scale-up to other low-barrier HIV settings? [specific to sub-set of service delivery stakeholders]

Alternatives to CoCM (in addition to CoCM, instead of CoCM, other ways to address behavioral health needs -> best practices, ideal, practical/feasible)?
