# Supplementary material for: Integrating behavioral health care into a low-barrier HIV clinic using the Collaborative Care Model: a mixed methods evaluation of patient care cascade outcomes and determinants
Source: Implement Sci Commun. 2025 May 5;6:53. doi: 10.1186/s43058-025-00738-5 (PMC12053849; doi:10.1186/s43058-025-00738-5)
Supplement: Supplementary file 4 — Additional file 4. Assessment of barriers and facilitators to implementing CoCM and factors associated with sustaining CoCM. [file 43058_2025_738_MOESM4_ESM.docx]

*Additional File 4: Assessment of barriers and facilitators to implementing CoCM and factors associated with sustaining CoCM*

**Barriers and facilitators to implementing CoCM at the Max Clinic:**

Staff hesitation around use of the PHQ-9 and the NIDA Quick Screen lay at the intersection of the culture of low-barrier care at the Max Clinic and existing familiarity with patient’s BH needs

Unlike in many primary care settings, the Max Clinic does not utilize a medical assistant to accompany patients to examination rooms nor does the clinic ask patients to fill out health questionnaires prior to or upon arrival at the clinic. Typically, a non-medical disease intervention specialist will accompany patients from the waiting area to the examination room, determine the patient’s goals for their visit, and initiate contact with social workers or physicians as needed to address those goals. In this context and based on feedback during the small group consensus meetings during the formative evaluation, we decided to administer the assessments in the screening (as well as intake and during CoCM care delivery visits) verbally. However, multiple staff members noted that “there's more of an emotional component when someone's asking you this [screener compared to the patient filling it out electronically or on paper]. I think it just hits differently” (Staff).

Measurement-based care is a core component of CoCM, especially for patients with depression and so routinely completing the PHQ-9 as a marker of tracking progress is essential to implementation. Yet completing this and the other components of the standardized screening (as well as the intake and care delivery visits) felt at odds with the culture of the Max Clinic according to some staff members.

*A lot of the reason that people like the Max Clinic is it feels like a family… You come in, you feel like you know people. And then when you switch to a standardized questionnaire, it feels really impersonal. It doesn't feel good. It feels kind of like suddenly there's this wall between you and the other person and they don't know you at all* (Staff).

Furthermore, Max Clinic staff members had extensive existing knowledge of their patient’s existing behavioral health needs. This knowledge precipitated the early-stage implementation adaptation to do a targeted screening led by social workers and physicians. But according to Max Clinic staff members, it also made the standardized screening components feel a bit redundant and insensitive.

*I had this [feeling] where there was a sense that we already knew a lot of these patients were depressed. It almost felt like turning the knife or something… “How often do you feel bad, feel like you're a failure to yourself and your family?” I mean, I don't know. That's the question that always sticks with me where I'm just like... when a lot of patients aren't in contact with their family. They don't even have a family or friends to feel bad about. It just felt like such... I don't know* (Staff).

When administering these standardized assessments in the context of existing knowledge and familiarity with the patient’s (often complex) socio-economic and personal histories, these tools came across to Max Clinic staff members as problematic or even potentially triggering (in a non-traumatic way) to patients. These challenges speak to CoCM’s compatibility within the existing Max Clinic workflows and culture.

In reviewing patient records, the PHQ-9 was almost universally completed while the NIDA Quick Screen was sometimes not, which some staff members attributed to it being more challenging to administer. “The NIDA felt almost harder to ask… I felt people were more reticent to disclose [substance use compared to] the PHQ-9” (Staff). Another possible explanation is because staff members were generally already aware of the substances endorsed by patients and therefore chose not to administer the NIDA Quick Screen due to the knowledge that the screening would not lead to an available intervention if they did not endorse using opioids.

*In a way, I get why we put [the NIDA Quick Screen] on there with all the different substances. It's good information to get and it kind of warms people up to the opioid question. But it also felt like “why are we asking these questions about other substances we're not treating [since] we're not offering interventions specifically for the other ones”* (Staff).

Another reason may have been confusion over the screening process and whether or not to complete the NIDA Quick Screen if they anticipated that the patient participant was only interested in care for depression or if they were only expected to advance through the depression cascade.

Some staff commented that their ability to properly phrase and introduce the standardized assessments, especially the PHQ-9 led to more patient willingness to be screened.

*I didn't really set it up too much. I just said, "I'm going to ask [you some] questions. Do you have five minutes of your time you can give me?" And when I asked it that way, they said "Yes," but when I [told] them that I'm going to do a screening questionnaire on depression, then it was like, "No, I don't have time…" Then I got more screening questions done* (Staff).

During the formative evaluation, some staff members highlighted the importance of how to frame and present CoCM with a focus on improving the program’s reach and this quote speaks about this. Although the Max Clinic staff have training in and are familiar with the use of such assessments, our multi-component implementation strategy (see Table 1) focused exclusively on training the care manager on use of assessments including the PHQ-9 and not the Max Clinic staff members in general.

Time and physical resource constraints were not as bad as anticipated by stakeholders, though they were still perceived to negatively impact CoCM’s feasibility

During the formative evaluation, staff members identified multiple resource-related constraints, such as room availability and short patient visits, that affected organizational readiness to implement CoCM. Room availability was mentioned prominently given that the Max Clinic only has three patient examination rooms and due to the unpredictable patient flow associated with the walk-in model, the clinic experiences patient backlogs when numerous patients arrive at the same time. The Max Clinic staff members observed that screenings, intakes, and CoCM care delivery visits could sometimes last longer than other patient visits. When these lengthy visits occurred during busy hours, they could exacerbate patient backlogs.

*That became an issue a number of times where [the care manager would] be doing an intake with somebody. And I think the intakes took a half an hour or something like that, where we would just be slammed out front and she'd be in one of the patient rooms and it would be this like, "Okay, so those are the times we need you to not be in one of the three Max [Clinic] rooms”* (Staff).

The observed length of screenings was also a factor that may have affected staff member uptake and buy-in of the program. In particular, the very first screening done after the program started took over 90 minutes to complete. The social workers recalled that each question led the patient into a larger, emotionally-charged conversation and they had trouble redirecting the patient back to the screening questions while also responding in an empathic way. “There was a worry that [a very long screening] would happen again and that, once again, it could take 10 minutes [or] it could take 90 minutes… I think that was a big worry for people, especially when there was a lot going on in the clinic, which often there was” (Staff). Unintentionally, this instance may have set the tone for staff member hesitancy to complete screenings during particularly busy times.

That said, this perception of availability was not universally shared by all patients. The engaged patients in general found that the care manager was readily available. “[There was] never a time where I came [to the Max Clinic] where she wasn't available… so I'd say that her availability was awesome” (Patient, engaged, depression). We captured in the REDCap database and in the care manager’s programmatic records the number of visits that patients made to the Max Clinic but during which they did not meet with the care manager. Among patients who were referred (n=42), there were 11 patients with recorded visits where they attempted to meet with the care manager but she was unavailable (e.g., she did not work that day or was seeing another patient), but no such visits among patients who completed an intake (n=27). Among referred patients there were four patients with recorded visits who declined to meet with the care manager who was available, but no such visits among patients who completed an intake.

Care manager self-efficacy in delivering Behavioral Activation was low, but concerns arose if it was the best choice of psychotherapy for low-barrier HIV care

During the preparation phase, the research team decided to adopt Behavioral Activation as an evidence-based psychotherapy to be used in conjunction with CoCM. As part of the multi-component implementation strategy, the care manager received training in Behavioral Activation during 1:1 skill building sessions with research team members. But quickly after the program entered the implementation phase and when presented with patients in the context of low-barrier care delivery, the care manager’s confidence to deliver Behavioral Activation dissipated.

*I had this training in Behavioral Activation and that was good. But then we actually started the program and it was like, "Oh, well, Behavioral Activation really isn't going to work that well because it's very systematic..." I didn't feel very confident in my skills doing that. I felt like I had to adhere very strictly* (Staff).

Like the other staff comments about the standardized nature of administering the PHQ-9 and other assessments, the care manager found the systematic nature of Behavioral Activation as they learned it in their training to sit at odds with the way low-barrier care at the Max Clinic was delivered.

*With most people, [I] never really got even started [with Behavioral Activation] because it's like you'd have the intake and then you wouldn't see [the patient] for a month… I'm not going to jump right into it because I feel like we need to use this time to catch up. “What's been going on in your life in the past month?" Then [the patient] only ha[s] 15 minutes to meet [with the care manager]… A lot of times they're going through a crisis, and it just felt like a mismatch to [do Behavioral Activation]... I felt like I needed to do mostly supportive counseling, like listening, and empathetic listening, and validation, and that kind of stuff. It just felt weird to then try to turn it to, "Well, what are some pleasant activities you can do this week? Can you take a walk three times a week”* (Staff)?

This was due to the gap between patient visits to the Max Clinic (due to its walk-in nature), the limited number of visits patients would have with the care manager, and the complex medical and BH needs that patients present to the clinic with.

Relatedly, another adaptation to a core component of CoCM that arose in response to the care manager’s self-efficacy to deliver Behavioral Activation in conjunction with the brief patient visits, and staff experiences using the PHQ-9 was to relax use of the PHQ-9 for patients with depression during each visit with the care manager. Like the adaptation to the screening, this represented an adaptation to a core component of CoCM (measurement-based care), but was deemed necessary by the care manager in conjunction with the Principal Investigator.

Modeling the Max Clinic’s culture facilitated the care manager’s engagement with patients

Max Clinic patients who interacted with the care manager, especially those at the engaged step who had either in-person CoCM care delivery visits or phone and text conversations, frequently cited the care manager’s warmth, availability, and approachability. These qualities were both mentioned in contrast to other BH services and providers through which they have previously received care and they were in sync with the culture of care delivery at the Max Clinic, which they appreciated and valued.

*[The care manager] was excellent. She provided really good [care]... I thought the service was great because it wasn't just a psychiatrist that just prescribed me stuff and told me to get out, basically. It was somebody that was there for me to talk to about everything. Actually, I think she was the first person I told everything that was going on with me openly and honestly, and it was really great having her* (Patient, engaged, depression).

*I don't normally talk to people about that, but I felt really comfortable whereas I was able to get really vulnerable and discuss stuff like that with [the care manager]… Trusting someone's hard [in and of] itself, so the fact that I was able to trust her, [that] was extremely helpful* (Patient, engaged, OUD).

Staff concurred and echoed these reflections. “[The care manager] was lovely and we were great to have her. She had her way with patients and she was very good at what she did” (Staff). These traits highlighted the importance of how patients felt welcomed, valued, and listened to.

**Factors associated with sustaining CoCM at the Max Clinic:**

The patient participants at the engaged step of the care cascade expressed their enthusiasm for the Max Clinic to continue offering CoCM based on the positive experience they had receiving care from the BH care manager on-site at the Max Clinic. However, to sustain CoCM at the Max Clinic a new care manager would need to be hired due to turnover at the position. Patients responded to the question about sustainability in the context of knowing there would need to be a new care manager and these patients located their enthusiasm within the context of the positive relationship they had with the previous care manager. On whether or not the Max Clinic should sustain CoCM, one patient remarked “Sure. I would definitely, definitely [sic] take a look at it and engage in it and see how it works for me” (Patient, engaged, depression/OUD). Another patient responded to the question about sustainment of CoCM as follows: “Yeah [it should be sustained, but] I think it would be interesting to see how it would differ from person to person [different care managers], and if it would be the same or different” (Patient, engaged, depression). Thus, identifying a care manager for the next phase of implementation who re-affirms these qualities will be important

Overall, there was a range of responses among staff members as to the question of whether CoCM should be maintained at the Max Clinic. Some staff members were very enthusiastic: “I think it helped some people tremendously, but I think there was an element… that it helped [a lot more] to some degree… I'd like to see how the program [runs] over a longer period of time” (Staff).

Another staff member was not supportive about sustaining the program, primarily grounding their rationale in CoCM’s fit given the burden of other severe mental health comorbidities and substance use disorders which were exclusionary criteria for participation and thus tempered CoCM’s reach.

*No, I don't think we should [sustain CoCM]. I don't think it reached enough people to be effective. And sure, it helped a couple [of] people here and there, but I don't think CoCM is going to help the Max Clinic patients engage [in] mental healthcare. [Instead,] I think honestly, just having a mental health practitioner here who is able to do things in a less restrictive way… and someone who can be here full time as well* (Staff).

Other staff were supportive of sustaining CoCM if certain adaptations like expanding the inclusion criteria for more comorbidities were made to further improve its contextual fit. Several staff expressed a common theme around CoCM needing to adapt to serve patients with additional comorbidities.

*I think we probably should continue [CoCM] and just expand it [to cover more comorbidities like schizophrenia] and accept that we don't have a strong evidence base. So what?... It does seem plausible to me that if we brought additional resources to bear from people who are more knowledgeable, even if it's imperfect and there's no trial to support it, that it would still be worth giving a shot… I think that what we need is, as close as we can come to it is comprehensive psychiatric services in the clinic* (Staff).

Additional suggested adaptations included: increasing the care manager’s availability to full-time; making the screening process less time-intensive, more streamlined, and accessible to patients who could not attend in-person; and having a care manager with more training and experience providing mental healthcare, including more training in a wider array of (brief) evidence-based psychotherapy interventions. Multiple staff members hoped that the next care manager could be in the role for a longer time so that they could establish better rapport with patients and in turn see if more patients would engage more.

*We weren't able to have more patients integrated with [the care manager]… because it took so long, just the whole process for [the care manager] to get to the point where she was seeing a patient consistently or talking to them consistently. So by the time we were able to see that effect for a few of the patients that she was able to connect with, she only had a couple of months left* (Staff).

Staff members recalled that intra-team communication about patient involvement in CoCM was initially slow during the early implementation phase when the care manager was adjusting to clinical workflows. But intra-team communication improved once the care manager started having 1:1 huddles with social workers and other team members to collaborate on individual patient treatment plans.

*[There was a patient who] at one point had come in having really severe suicidal ideation and had a plan in place, just all the things lined up. And [the care manager and the social worker] partnered together in terms of assessing that situation, making a plan, following through with that plan. And I just thought it was really beautiful how the two of them supported each other and supported [the patient] through that. [It] would be helpful to [have] started [doing this] earlier* (Staff).

Multiple staff members discussed the importance of keeping up and improving upon these forms of communication to sustain CoCM. Thus, such forms of communication would continue sensitizing staff to the program and could serve to improve collaboration.
